# On Error-Related Potentials During Sensorimotor-Based Brain-Computer Interface: Explorations With a Pseudo-Online Brain-Controlled Speller

**DOI:** 10.1109/OJEMB.2019.2962879

**Published:** 2020-02-14

**Authors:** Michele Bevilacqua, Serafeim Perdikis, José del R. Millán

**Affiliations:** ^1^ Defitech Chair of Clinical NeuroengineeringCenter for Neuroprosthetics and Brain Mind Institute, EPFL CH-1202 Geneva Switzerland; ^2^ Brain-Computer Interfaces and Neural Engineering Laboratory, School of Computer Science and Electronic EngineeringUniversity of Essex 2591 Colchester CO4 3SQ U.K.; ^3^ Department of Electrical and Computer Engineering & Department of NeurologyThe University of Texas at Austin 12330 TX 78712 USA

**Keywords:** Brain-Computer Interface, error correction, Error Potentials, Motor Imagery, hybrid BCI

## Abstract

*Objective:* Brain-computer interface (BCI) spelling is a promising communication solution for people in paralysis. Currently, BCIs suffer from imperfect decoding accuracy which calls for methods to handle spelling mistakes. Detecting error-related potentials (ErrPs) has been early identified as a potential remedy. Nevertheless, few works have studied the elicitation of ErrPs during engagement with other BCI tasks, especially when BCI feedback is provided continuously. *Methods:* Here, we test the possibility of correcting errors during pseudo-online Motor Imagery (MI) BCI spelling through ErrPs, and investigate whether BCI feedback hinders their generation. Ten subjects performed a series of MI spelling tasks with and without observing BCI feedback. *Results:* The average pseudo-online ErrP detection accuracy was found to be significantly above the chance level in both conditions and did not significantly differ between the two (74% with, and 78% without feedback). *Conclusions:* Our results support the possibility to detect ErrPs during MI-BCI spelling and suggest the absence of any BCI feedback-related interference.

## I. Introduction

Brain-Computer Interfaces (BCIs) are gradually establishing themselves as a viable solution for replacing functionality in paralysis [1]. Motor Imagery (MI), where subjects employ imagined movements modulating cortical sensorimotor rhythms (SMRs), is a popular mental strategy for BCI applications because it offers the possibility of self-paced control through non-invasive imaging like electroencephalography (EEG) [2]. However, MI BCI does not ensure perfect decoding accuracy, which creates a need for error-correction mechanisms. This problem is conventionally addressed by exploiting secondary control modalities in a hybrid fashion [3], [4], by reserving active MI tasks for error-correction [5] or by embedding error-handling directly into the user interface [3], [6], [7]. These approaches suffer important shortcomings. Hybrid BCIs require residual abilities often unavailable to end-users. Reserving MI tasks for “undo” functionality is detrimental given the small number of mental tasks that can be usually decoded, while forcibly adding extra MI tasks reduces the overall accuracy, leading to additional errors. Lastly, error-correction mechanisms accessible through the main BCI modality (for instance, a “backspace” option) are themselves subject to the limitation of imperfect decoding [3].

It has been early realized that the possibility to detect endogenous error-related activity could avoid all the aforementioned drawbacks. Neuromarkers of error processing may be identified in the same signal used for the main BCI modality, leaving the latter completely unaffected. Fortunately, such EEG correlates, referred to as error-related potentials (ErrPs), have been already well studied. Specifically, an ErrP is an event-related potential (ERP) elicited by the neural processing of an erroneous event [8]–[10]. ErrPs are time-locked to the error onset, originate at the anterior cingulate cortex (ACC) and propagate to fronto-central scalp regions creating typical waveforms [10].

Despite MI and ErrPs have been individually thoroughly analyzed, applications combining MI control with ErrP detection are scarce. The first work arguing in favour of this possibility [11] is limited by the inclusion of only two subjects and the absence of a realistic scenario. In [12], a system is proposed where ErrP generation does not immediately follow MI commands and relies on additional feedback, thus probably closer resembling “observation”, rather than “interaction” ErrPs [13]. A hybrid MI/ErrP BCI to control a robotic arm was proposed in [14]. MI was used to select pre-determined robot movements. Subjects were subsequently assessing the robot's position relative to the target. ErrPs detected upon overpassing the target would stop the robot close to the desired position. There, engagement to MI and elicitation of ErrPs are separated by a lengthy, “effortless” observation task. Overall, we posit that these studies, notwithstanding their substantial contributions, have not adequately evaluated possible interference between MI execution and ErrP elicitation likely to occur in asynchronous applications, involving high cognitive workload and fast interaction. Furthermore, studies of “continuous” error-related activity [15] are currently inconclusive regarding applicability, and combinations of ErrPs with stimuli-driven paradigms [16], [17] can only support synchronous control.

This work studies ErrP generation while subjects assume they are in control of the BrainTree MI-based speller [3], testing the hypothesis that a hybrid approach with MI as the main control modality and ErrP reserved for error-handling is feasible under conditions of high mental and temporal demand. Ten subjects, including an end-user, are instructed to operate the speller with a 2-class MI BCI. The correctness of a speller action needs to be assessed immediately after the latest command, while subjects are still essentially engaged into MI. Error assessment requires substantial mental effort, as it involves a non-trivial process of interpreting the resulting new arrangement of multiple visual elements of the speller's graphical user interface (GUI). Additionally, control is quickly returned to the MI modality, forcing the user to quickly deciding the required type of the next MI task/command. Taken together, the chosen framework accurately represents the hurried and stressful conditions prevalent in a real-world setting.

We further address a second important issue. It is standard practice in MI BCI to provide continuous feedback, so as to help subjects maintain or improve their performance [2]. However, it is reasonable to assume that continuous BCI feedback might influence the generation of ErrP waveforms and, consequently, the ability of the system to detect them. That is because such feedback may effectively “warn” users of an upcoming error, removing the “oddball” element thought to be critical for the elicitation of an ErrP [8]–[10], or invalidating its expected time-locking to the actual error onset. To investigate this issue, the experiment was repeated for each subject in two conditions under a randomized, cross-over design: with (*FBon*) and without (*FBoff*) visual BCI feedback.

Our results offer substantial evidence for answering both research questions considered. The average ErrP detection accuracy was found to be significantly above the chance level and typical ErrP signals could be identified in both experimental conditions, suggesting that establishing a hybrid MI BCI application enjoying seamless, endogenous error-handling through ErrP detection is possible even in a highly demanding setting. In addition to this, the ErrP detection rate did not significantly differ between the two conditions (74% with and 78% without feedback), implying that the presence of visual BCI feedback does not hinder the elicitation and detection of ErrPs.

Given its exploratory nature and the need to establish feasibility before testing a fully closed-loop MI BCI system with ErrP-based correction, this study is limited in two ways: First, ErrP detectability is only attempted “offline” (automatic error-correction is imposed during spelling). Second, taking into account that current literature strongly implies the need for infrequent errors in order to extract high-quality ErrP signatures (“oddball” paradigm), and that this factor should be controlled for in order to test our hypotheses, the MI BCI component is replaced by a surrogate, pseudo-online controller—a fact participants were not made aware of, so as to invest the mental effort anticipated in a realistic scenario—allowing to fix the error rate to 20%. The subjects’ self-reports and the analysis of acquired MI data proves that users remained fully engaged with the requested MI task, validating this approach.

## II. Results

### A. ErrP detection During Pseudo-Online MI BCI Spelling

**Figure 1.**
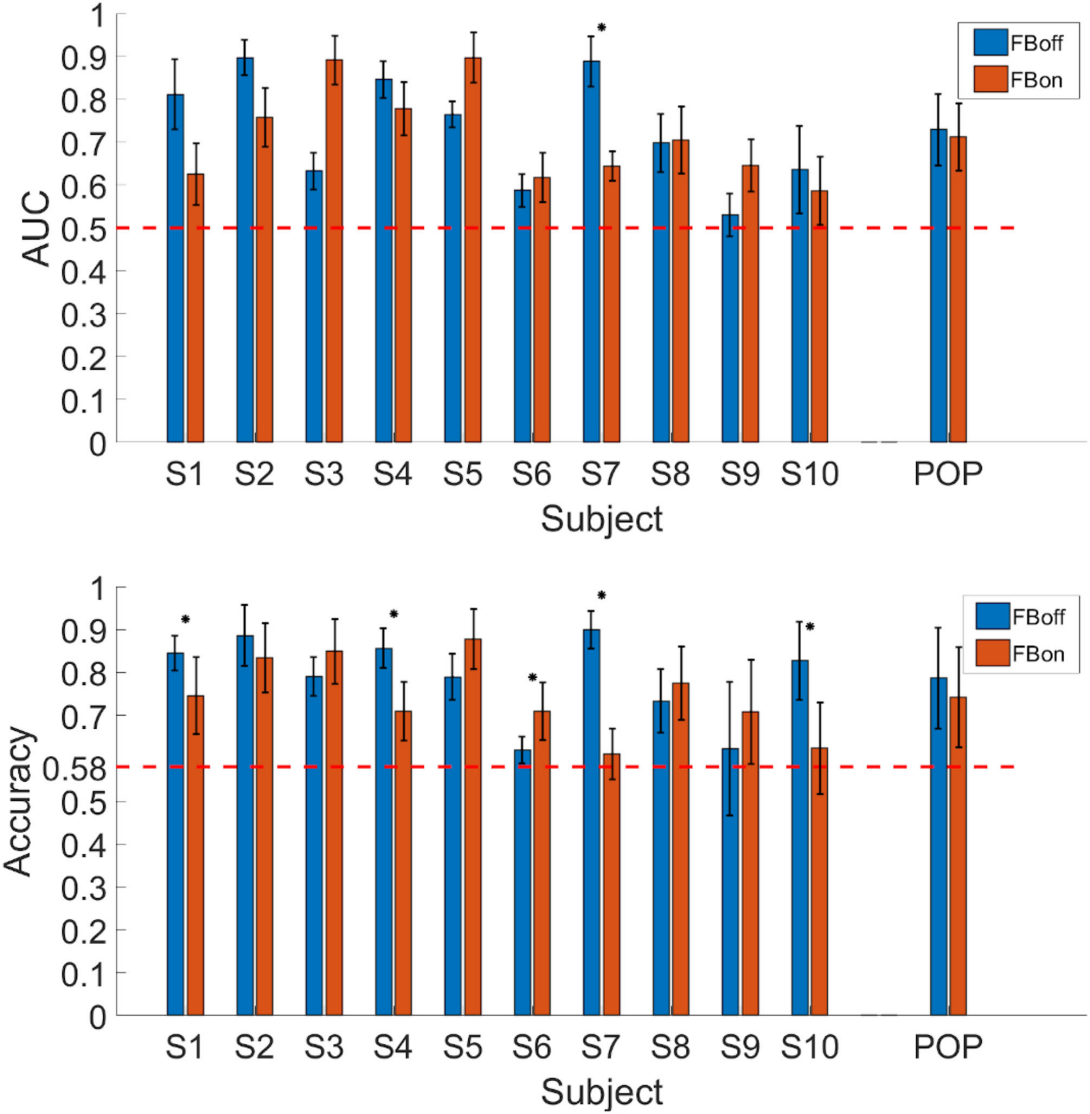
AUC (top) and Accuracy (bottom) of ErrP detection for each subject and condition, with population averages. The bars and errorbars of single subjects represent the means and standard deviations across cross-validation iterations, respectively. For population averages, errorbars illustrate the standard deviations across subjects. The horizontal red dashed lines visualize the theoretically expected performances of random classifiers. Asterisks above a pair of bars denote significant differences between the two experimental conditions at the 95% confidence interval (Wilcoxon paired signed-rank test).

Fig. 1 summarizes the main results of this study, namely, the Area Under the Curve (AUC) and classification Accuracy of ErrP detection. Regarding the main hypothesis, the average (across subjects) AUC and Accuracy for both conditions approaches or exceeds 0.7, thus clearly indicating the possibility of using endogenous error-related activity as an error-correction mechanism during EEG-based MI BCI spelling. Importantly, the individual participant results point to the same conclusion, as performances exceed those anticipated by a random classifier: AUC above 0.5 and classification accuracy over 0.58 (95% confidence interval for *N > 100* samples with binomial testing). Subject S9 in the *FBoff* condition is the only exception.

**Figure 2.**
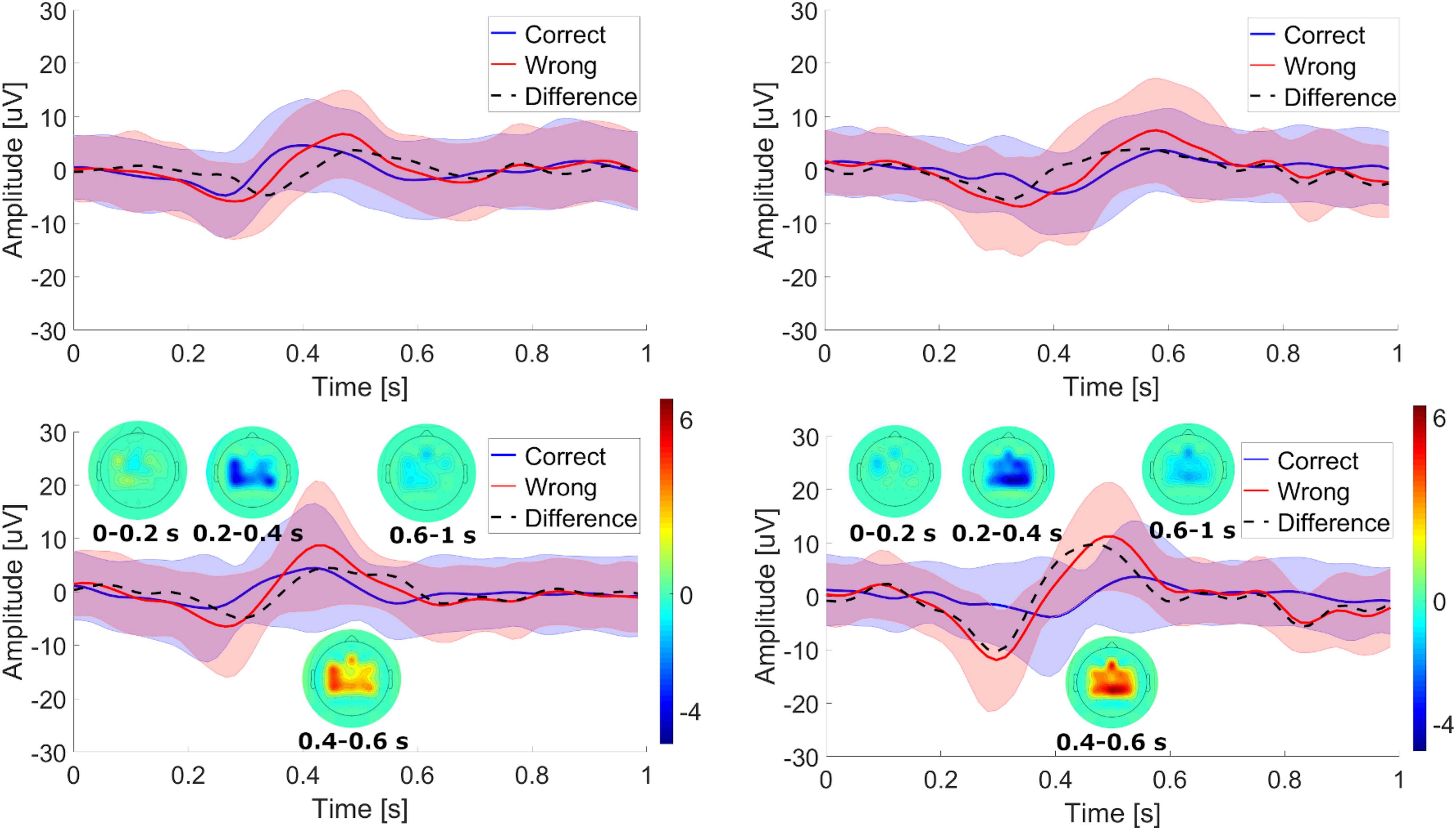
Grand average plots of correct and wrong ErrP epochs for subject S2 and channel CPz in the *FBon* (left) and *FBoff* (right) condition, before (top) and after (bottom) re-alignment. Time 0 corresponds to the end of a preceding MI trial. The shadowed areas represent the standard deviation across the single trials averaged. The topographic plots illustrate the scalp potential difference between wrong and correct epochs averaged in the period of time specified below, color-coded as shown in the colorbars.

The possibility of ErrP detection is further substantiated by the identification of typical interaction ErrP signatures in the data of all participants. Taking the example of subject S2, Fig. 2 shows the grand average ErrP waveforms (and their standard deviation) on channel CPz of the international 10–20 EEG placement system for conditions *FBon* (left) and *FBoff* (right), with (bottom) and without (top) a realignment procedure applied to ErrP epochs (see Section F in Supplementary Materials). As expected, the ErrPs (red) exhibit an early negativity around 250 ms followed by a positive peak around 500 ms in both cases. On the contrary, epochs following a correct MI command (blue) are distinctly flatter. Furthermore, ErrP activity is localized on the fronto-central and parietal areas monitored, consistent with a signal known to generate in deeper cortical structures and to spread radially over the scalp. The beneficial effect of ErrP realignment is evident in Fig. 2.

**Figure 3.**
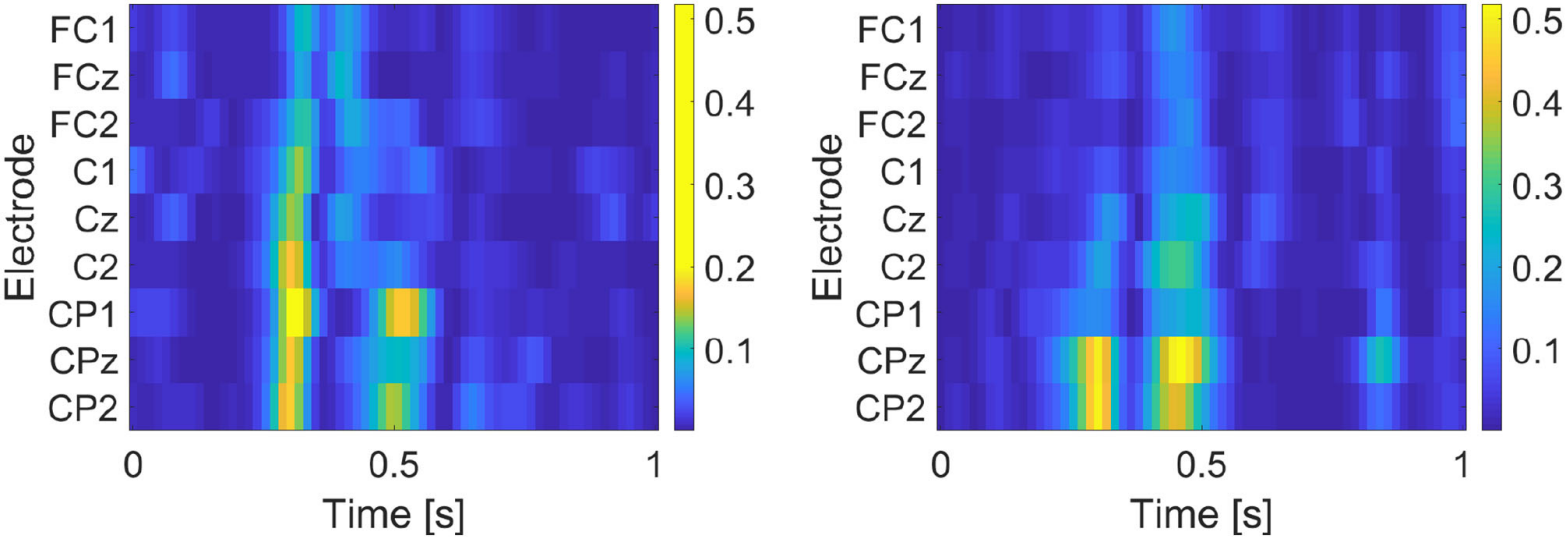
Fisher Score of the ErrP features of subject S2, for the *FBon* (left) and *FBoff* (right) conditions on the subset of channels considered for decoding. Time 0 corresponds to the end of a preceding MI trial.

A visual representation of the candidate features' Fisher Score for subject S2 in the two conditions, averaged over the 6 folds of cross-validation (data of each word spelled form the respective fold), is illustrated in Fig. 3. As anticipated, the most relevant features (bright color) correspond to the time points associated with the negative and positive peaks of the ErrP grand averages in Fig. 2, as well as on the channels where these peaks are more prominent. Hence, there exists enough discriminant power in the identified ErrP signals to justify the favorable classification results (Fig. 1).

### B. Effects of Visual MI BCI Feedback on ErrP Detection

Regarding our second hypothesis, comparing the two experimental conditions in Fig. 1 determines that the magnitude of the MI BCI feedback provision effect on ErrP detection is, on average, negligible and statistically insignificant (*p = 0.58*, Wilcoxon paired signed-rank test across subjects for the two conditions). On the individual subject basis, the majority of participants exhibited similar performances in both conditions. Subjects S4 and S7 performed slightly better in the *FBoff* condition, however, the difference was statistically significant only for S7 and only with respect to classification accuracy (*p = 0.0313*, Wilcoxon signed-rank test, where cross-validation iterations compose the test's samples). Subject S9 was the only one performing substantially better with the *FBon* condition. Overall, ErrP detection performance seems to be largely independent of providing visual MI BCI feedback.

## III. Discussion

This work has investigated, first, whether ErrP detection from EEG is possible while subjects engage into self-paced control of a cognitively demanding application. Confirming this hypothesis paves the way for embedding seamless and efficient error-correction mechanisms into SMR-based BCI applications. Second, we set out to determine whether the presence of MI BCI feedback interferes with the elicitation of ErrPs. Our results provide substantial evidence towards confirming the first hypothesis and rejecting the second.

Specifically, the AUC and classification accuracy of ErrP detection were found to be, on average, significantly above those of a random classifier (AUC/Accuracy of 0.72/0.78 for *FBoff* and 0.71/0.74 for *FBon*), as well as for all participants individually (Fig. 1). These figures are in line with the results obtained in the literature of ErrP classification for similar dual tasks in less demanding situations [12], [15] or even in non-hybrid scenarios, where error monitoring is the subjects' only task [8]–[10]. In addition to that, the derived electrophysiological dynamics of ErrP generation in both conditions were highly consistent with the prototypical waveforms of “interaction” ErrPs described in the literature [8], [10]. Therefore, it seems that immediate correction of errors committed by another (main) BCI control modality is possible, lifting the limitations of “work-around” methods that have been exploited so far. On the downside, these average performances, or even the best recorded performance achieved by subject S2 in condition *FBoff* (AUC/Accuracy of 0.89/0.88), are still far from perfect. This means that ErrP-based error-handling may contribute to improved BCI control, but it still cannot stand as the sole error-handling mechanism available. In the example of a speller, as used here, a “backspace” functionality should still be provided as a last resort.

Concerning the second hypothesis, our results strongly advocate the absence of any effect of providing continuous MI BCI feedback or not on the elicitation of ErrPs. The difference in AUC and Accuracy between the two conditions (Fig. 1) was marginal and not statistically significant, while no substantial differences in EEG signatures of generated ErrPs could be spotted (Fig. 2 and 3). Only for subject S7 the difference in accuracy between the two conditions was significant (*FBoff* 0.89, *FBon* 0.61). Five subjects (S1, S2, S4, S7, S10) performed somewhat better with the *FBoff* condition, while another three (S3, S5, S9) with *FBon*. For two subjects (S6, S8) the result was ambiguous. This implies that MI-based BCI application designers that wish to incorporate ErrP-based error-handling may do so without having to remove the common feedback accessory supporting the user's MI control.

The suspicion that continuous MI BCI feedback may interfere with ErrP elicitation was grounded on the fact that such feedback effectively warns the user for an upcoming erroneous command, removing the element of surprise from the brain's error-realization processes. The latter (i.e., a discrepancy between the predicted next interface action and the actual feedback) has been thought to be an important factor shaping the interaction ErrP waveform. It is for this reason that most ErrP generation protocols adopt oddball paradigms: infrequent errors are bound to be less predictable. Hence, the absence of an effect in this work adds up to recent evidence suggesting that the necessity of an oddball ErrP paradigm might have been exaggerated [9]. Instead, the typical interaction ErrP signature in EEG may mainly represent the realization of a mismatch between the user's intention or projected optimal outcome and the actual interface action, since this incongruence is preserved even with strong evidence for an imminent error. Indeed, in our experiment, although the BCI error rate was fixed (20%), in the case of an erroneous BCI outcome the continuous BCI feedback was well above this error rate and yet an ErrP was elicited and detected. It should be highlighted that not having to rely on oddball paradigms in order to retrieve ErrPs of good quality is critical for substantiating the first hypothesis in the most realistic circumstances, i.e., when the speller is operated with real closed-loop MI control and ErrP detection. MI performance, which is known to be fairly unstable, may (at least temporarily) lead to frequent errors. It is exactly in these situations that accurate ErrP detection can be particularly useful to improve BCI performance and user experience.

It could be argued that replacing closed-loop with pseudo-online MI control through “playback” trials invalidates our claim of testing a realistic and cognitively demanding hybrid BCI scenario, even if participants were unaware of this manipulation. To alleviate such concerns, we performed open-loop MI-BCI analysis (see Section G of the Supplementary Materials) showing that all but one subjects exhibited neurophysiologically sound SMR patterns and above-chance classification MI accuracy, confirming that they were immersed into the main BCI task, as with a real BCI. Furthermore, no subject declared realization or suspicion of not being in control when asked.

Building upon the results extracted in this work, its natural extension and ultimate goal of this line of research is to reproduce it in a fully online setting, where subjects control the interface by means of their spontaneously emerging SMR patterns and where ErrP-based error-correction is enabled in real time, automatically canceling mistaken MI commands. This will allow an experimental evaluation of the improvements in efficiency and effectiveness that can be achieved thanks to ErrP-based error-correction in comparison with our previous work [3]. Future work could also investigate the value of ErrP decoding in MI BCI adaptation, since the elicitation of error-related activity implicitly provides clues about the MI task currently executed by the user. This contextual information can be exploited for adapting the MI BCI classifier as proposed in [18]. Last but not least, it would be interesting to study the EEG correlates of “continuous” error processing elicited while subjects are observing the MI BCI feedback, as well as to investigate ways of exploiting such signals for correcting spelling mistakes.

## IV. Conclusions

Concluding, this work substantiated the possibility of enabling ErrP-based spelling mistake corrections while subjects believe to be in control of a BCI text-entry system through MI. It has also showed that the provision of MI BCI feedback does not prevent the elicitation of typical interaction ErrP signals. Our study has provided the foundations for implementing a closed-loop MI BCI spelling application embedding automatic error-correction through the user's own endogenous activity.

## V. Materials and Methods

**Figure 4.**
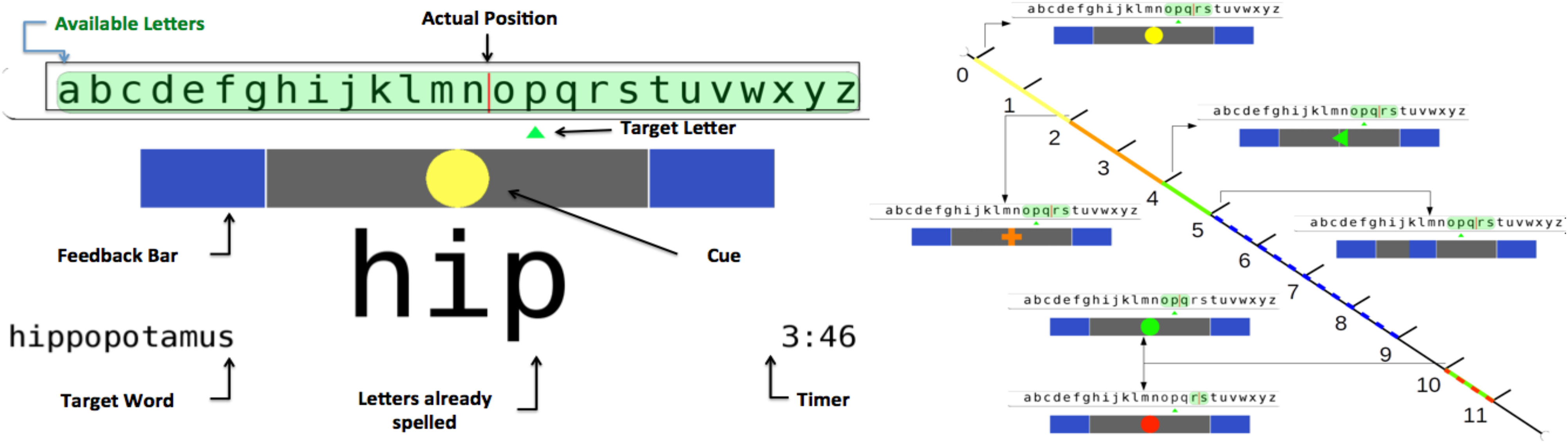
Left: BrainTree GUI arrangement at the beginning of a character selection trial aiming to spelling the letter “p”. Right: Spelling protocol trial structure with associated visual cues.

The BrainTree speller, employed as a testbed BCI application exerting high mental and temporal workload, is elaborately described in [3], [18]. Subjects were instructed to engage into a pair of MI tasks of their choice (among left/right hand and feet imagery) to push the speller's cursor left or right towards the desired character, until the latter was typed (Fig. 4 (left)). Participants were unaware of the fact that actual speller control was artificially generated by “playing back” a randomly chosen trial from a collected database of 300 instances derived in [3], thus manipulating the MI command error rate to about 20%. The study complied with the declaration of Helsinki and recruited 10 participants who signed informed consent. EEG was acquired with a g.USBamp amplifier (g.Tec medical engineering, Schiedelberg, Austria) and 16 active electrodes over the users' fronto-central cortex (10-20 system). Users were asked to spell 6 words with (*FBon* condition) and without (*FBoff*, feedback bar removed from speller GUI, Fig. 4 (left)) MI feedback. The order of conditions was randomized across subjects. The trial timeline is illustrated in Fig. 4 (right). ErrP onsets were assumed adjacent to the end of the preceding MI trial, abruptly moving the speller's cursor left/right. Artificial MI BCI control was implemented by applying the MI BCI methods of [2], [3] (Laplacian spatial filtering, Welch-method power spectral density feature extraction and selection by discriminant power ranking, classification with a Gaussian framework, sample rejection, evidence accumulation and decision thresholding) on the “playback” trials. The same basic methods are applied for extracting offline MI BCI results, except for adopting a Linear Discriminant Analysis model for classification. ErrP epochs were filtered in 1-10 Hz (4^th^ order IIR), downsampled to 64 Hz and classified with LDA using cross-validation. Before classification, ErrPs were realigned with a method iteratively optimizing the cross-correlation of single epochs to the grand average. Materials and methods are elaborated in the Supplementary Materials.

## Supplementary Materials

The Supplementary Material provides a detailed description of Materials and Methods, as well as the analysis of the MI-BCI.


