# Supplementary material for: On Error-Related Potentials During Sensorimotor-Based Brain-Computer Interface: Explorations With a Pseudo-Online Brain-Controlled Speller
Source: IEEE Open J Eng Med Biol. 2020 Feb 14;1:17–22. doi: 10.1109/OJEMB.2019.2962879 (PMC8979633; doi:10.1109/OJEMB.2019.2962879)
Supplement: Supplementary file 1 [file supp1-2962879.pdf]

Supplementary Materials

On Error-related Potentials during Sensorimotor-based Brain-Computer Interface: Explorations with a Pseudo-Online Brain- Controlled Speller

Michele Bevilacqua, Serafeim Perdikis, *Member, IEEE* and José del R. Millán, *Fellow, IEEE*

A. BrainTree speller

To probe our hypotheses, we have adopted the "BrainTree" speller employed also in our previous work [1], [2]. BrainTree's GUI is shown in Fig. 4 (left). At the top, an ordered alphabet is displayed. The current position is represented by a red cursor. Characters available for selection are included in a light green "bubble". The cursor moves in either direction at the end of every MI trial depending on the outcome of the latter (left/right), taking a new position closer to the user's desired character, while the green bubble updates accordingly. The speller's underlying structure, described in detail in [1], decides the final position of each transition ensuring that probable next characters are reachable within a few steps. A letter is selected when it is the only one remaining within the green bubble in the direction of the last cursor movement. As a visual aid, a green arrow was always pointing to the target letter. Below the character bar, the GUI displayed a conventional MI BCI feedback bar and the protocol's cues (see Section B). At the GUI's bottom left corner, the complete target word was displayed, while at the bottom center the letters already spelled by the user were shown. A timer at the bottom right corner was illustrating the time spent so far in the current spelling task.

B. Control paradigm

The binary speller was controlled by means of a pair of MI tasks, one of which would move (once successfully detected) the speller's cursor to the left and the other to the right. The options given to the participants were right hand, left hand and both feet MI. Every subject could choose the 2-class taskset (right hand/left hand, right hand/both feet or left hand/both feet) they felt more comfortable with. The "hand-based" MIs would always cause the speller's cursor to move towards the respective direction (i.e., left hand MI to the left).

Subjects were told they were actively controlling the speller through a closed-loop MI paradigm. Experienced subjects were instructed to adopt the taskset they were known to be most successful with and were informed that their last trained classifier was used. Naive subjects were instructed to adopt any of the available tasksets they felt more comfortable with after a short mental rehearsal, and were informed that a corresponding subject-unspecific classifier trained on previously collected MI datasets would be used.

However, actual control of the speller was in fact artificially generated. Every new trial was a "playback" of a randomly chosen trial from a collected database of 300 instances derived by the study reported in [1]. This design choice has been taken to allow a fixed 20% percentage of erroneous trials throughout the experiment for all subjects, thus supplying an adequate number of errors to power subsequent statistical analysis, while maintaining the "oddball" element thought to be crucial for the elicitation of ErrPs. Finally, the MI calibration phase for naive subjects could be skipped, greatly relaxing the logistics of the experiment. Importantly, this ``playback" method replicated not only the accuracy and command delivery timing of an expert MI BCI user, but also the usual dynamics of the BCI's decision making process as depicted in the MI feedback (see Section E), so that the absence of actual MI control was not distinguishable even by the most experienced subjects. To verify the latter, the manipulation trick was revealed to each participant after the experiment and they were asked to report whether they harbored any suspicion of not actually being in control of the interface at any time during the spelling sessions.

C. Experimental paradigm and trial structure

An experimental session consisted in spelling the following 6 words (always in this order): *hippopotamus*, *wolf*, *jellyfish*, *turtle*, *leopard* and *reindeer*. These words were chosen for requiring a balanced number of left and right trials in order to be correctly typed. Every subject performed

two sessions. In one of the sessions the spelling tasks should be accomplished with (*FBon*) and in the other without (*FBoff*) MI BCI feedback. In the latter case, the GUI's MI feedback bar was disabled (not shown), while all other GUI elements remained identical. The order of sessions was randomized across subjects, so as to mitigate learning and habituation effects that could bias their comparison. Five randomly selected subjects (S2, S5, S6, S9 and S10) started with the *FBon* condition and another five (S1, S3, S4, S7 and S8) with *FBoff*. Each session lasted approximately 1 hour.

Fixing the percentage of mistakes to 20% allowed to obtain approximately 45 erroneous and 180 correct trials for every subject and condition. The onset of the elicitation of an ErrP waveform was taken to be the moment of the movement of the speller's cursor at the end of an MI trial. In case the cursor moved in the intended direction, the one corresponding to the MI task just performed, the trial would be labeled as “Correct”, otherwise, as “Error”. Correct commands would lead to the associated updates of the GUI [1]. In the event of a wrong command, the speller would automatically “undo” it after 1 sec (so as to not interfere with ErrP elicitation) by restoring the GUI's previous state. Each new trial consisted of 5 consecutive states indicated by visual cues superimposed on the speller's GUI for both conditions, as shown in Fig. 4 (right): i) Relax/Pause (1s) ii) Fixation (2s) iii) Task indication (1s) iv) MI epoch (up to 4s, timeout imposed) v) Correctness feedback (1s). The task and correctness cues were provided to facilitate users in the early phases of the experiment, when still not accustomed to the speller. Users were supposed to have already deducted the respective information by interpreting the GUI elements' arrangement, i.e., the position of the red cursor with respect to the desired character and the inclusion of the latter (or not) in the green bubble.

D. *Experimental setup and participants*

Ten subjects (8 male, age 27.3 ± 3.5 years) participated in the study. Nine subjects were able-bodied with no known neurological conditions and intact or corrected vision, while subject S1 was suffering from Spinal Cord Injury (SCI, ASIA A). All subjects signed informed consent and the study was conducted in accordance with the declaration of Helsinki. Six subjects (S1, S2, S3, S4, S5 and S6) were experienced with MI BCI, while the remaining four were BCI naïve users.

EEG activity was recorded at 512 Hz sampling rate using a commercial g.USBamp biosignal amplifier (g.Tec medical engineering, Schiedelberg, Austria) with 16 active electrodes distributed according to the extended 10/20 international system so as to adequately cover the user's sensorimotor and fronto-central cortices (Fz, FC3, FC1, FCz, FC2, FC4, C3, C1, Cz, C2, C4, CP3, CP1, CPz, CP2, CP4) with the ground on AFz and the reference on the right earlobe. During the spelling sessions, participants were comfortably seated on a chair approximately half meter away from a computer screen where the visual protocol was displayed. The SCI end-user participant was seated in his own manual wheelchair.

E. *Pseudo-online MI BCI*

The MI BCI scheme described in [1], [3] was adopted in its entirety (Laplacian spatial filtering, PSD feature extraction and selection, classification with a Gaussian framework, sample rejection, evidence accumulation and decision thresholding) to give subjects the feeling of control over the speller. As in [1], the “integrated” probabilities, the output of the evidence accumulation module, were driving the decision making and the feedback bar visualization during the MI epoch of each trial. The latter was only shown in the *FBon* condition, so that the distance of the bar's blue “liquid cursor” from either edge of the bar would inform the user how close he/she is to delivering the corresponding MI command (Fig. 4 (left)). The only difference has been feeding to the BCI algorithms the raw signals from a selected trial in the collected database (which was recorded with the same EEG setup), instead of the actual ones recorded in real time from the user's scalp. The “playback” trials were selected as follows: First, the algorithm would randomly decide whether the next trial would be correct/wrong with probability 0.8/0.2, respectively. This automatically also defined whether the next trial should result in a left or right transition, based on the current state of the speller. Finally, a trial with the determined feature combination (e.g. “wrong left”, “correct right”, etc.) was randomly loaded from the database and fed to the MI BCI processing pipeline.

F. *Data analysis methods*

1) *Motor imagery analysis*

Analysis on the subjects' MI BCI aptitude has been performed in order to validate our assertion that subjects remained unaware of the pseudo-online nature of the MI paradigm and engaged meticulously into the MI tasks. EEG were spatially filtered using a Laplacian filter, DC was then removed and the data were band-pass filtered between 1 and 50 Hz using a linear phase IIR Butterworth filter of 4th order. Identically to the pseudo-online MI BCI, PSDs of the epoched signal in the frequency range 8-30 Hz have been computed using the Welch method. MI performance was estimated for each subject and condition using 6-fold cross-validation, where each fold corresponded to the data of one of the 6 words in the spelling protocol. Classification accuracy was extracted by means of LDA models. Feature selection (using only the training folds of each cross-validation iteration) has been performed by ranking candidate features according to their Fisher Score discriminant power index and selecting automatically the 10 best, in this aspect, features to train an LDA classifier and apply it on the testing fold. A final performance estimate is reported as the average classification accuracy across the 6 cross-validation iterations.

2) *ErrP analysis*

For ErrP analysis, EEG data were filtered between 1 and

10 Hz using a linear phase IIR Butterworth filter of 4th order. No spatial filtering has been applied. The data were then downsampled to 64 Hz. The beginning of each ErrP epoch coincided with the end of the corresponding MI epoch, i.e., the moment at which the speller's cursor and the green bubble update their state. The expected duration of the ErrP epochs is 1 sec [4], [5].

A re-alignment procedure was applied on each ErrP epoch in order to cope with potential inconsistencies of the ErrPs' time-locking to the considered error onset. Specifically, an iterative approach has been implemented, where each single epoch was shifted by a certain time interval so as to maximize the cross-correlation between itself and the grand average epoch waveform. The latter was updated with the realigned epochs and the overall process was repeated until a new iteration would not bring about any further realignment. The maximum allowed shift was fixed to 500 ms (forward or backward). It should be noted that allowing forward search in the application of this realignment framework to the real-time, closed-loop setting, would only delay ErrP-based corrections for maximum 500 msec, a fact that we view as minimally detrimental to the user experience. For ErrP detection evaluation, 6-fold cross-validation was applied, where each fold comprised the data of a single spelling task (word). The candidate features were the 64 time samples of each trial on 9 selected channels: FC1, FCz, FC2, C1, Cz, C2, CP1, CPz and CP2). Fisher Score was used to rank the features.

For each cross-validation iteration, LDA models where trained for 7 different numbers of features used, NF, from 5 to 35 with a step of 5. LDA estimation involved standard covariance shrinkage, whose parameter ρ was also optimized considering values from 0 to 1 with a step of 0.05. Receiving Operating Characteristic (ROC) curves and the corresponding Area Under the Curve (AUC) have been computed for each subject and condition. The reported AUCs correspond to the average ROC curves across the cross-validation iterations derived for the optimal tested combinations of the two hyperparameters considered, NF and ρ. For these values, we also report a classification accuracy result for the point on the ROC curve that maximizes the average of true positive and true negative rates (i.e., the point that equally punishes both error types).

G. *Open-loop MI analysis results*

The Fisher Score values of candidate MI Power Spectral Density (PSD) features for subject S2 and their spatial distribution within the μ (8-14 Hz) and β (18-24 Hz) frequency bands in the two conditions are reported in Supp. Fig. 1. This subject showcases typical sensorimotor rhythm (SMR) modulations for the left/right hand MI taskset employed, specifically, contralateral (to each hand imagination) activations constrained in the expected μ and β bands. Adequate modulability of SMR brain patterns could be found for all participants, except for S1.

The single-sample classification accuracy averaged over the cross-validation iterations for each subject (Supp. Fig. 2) confirms that all participants, except for S1, were able to maintain an adequate level of MI control, which greatly outperformed that of a random classifier. The latter is expected to reach at best a 0.58 classification accuracy (just by random chance) at the 95% confidence interval for the number of samples available in our study. Hence, these results support that despite the pseudo-online MI BCI implemented here, subjects were fully engaged into what they thought is online MI control of the spelling interface. Furthermore, all subjects reported in a post-experimental questionnaire that they had no suspicion of not being in control of the speller at any time during the spelling sessions.

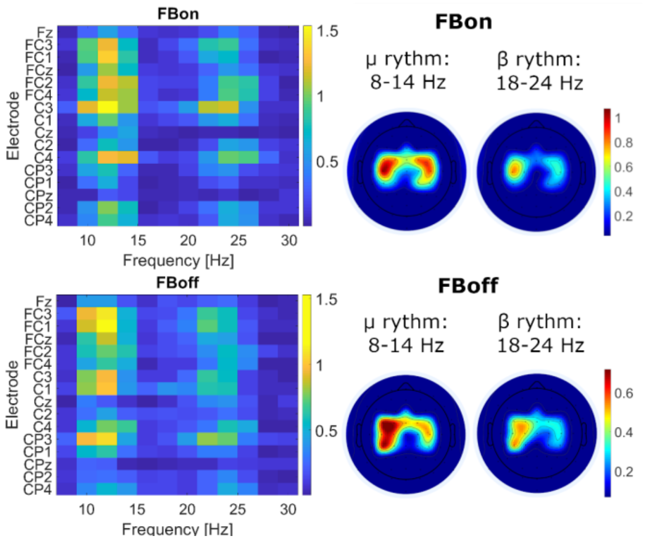

Supp. Fig. 1. Fisher Score of candidate MI PSD features across all channels and frequency bands for subject S2 and both conditions (left). Spatial distribution of MI feature Fisher Score values averaged within the μ (8-14 Hz) and β (18-24Hz) bands for both conditions (right).

It is worth to mention that, although there does not seem to be an effect of feedback provision on MI performance here, this should be anticipated given that the provided feedback was artificial and the overall training time in this experiment was too short to induce operant learning.

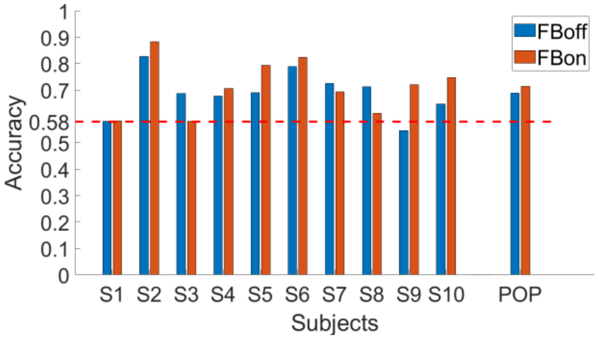

Supp. Fig. 2. Single-sample classification accuracy of MI patterns for all subjects. The horizontal red dashed line denotes the theoretically expected performance of a random classifier.

REFERENCES

[1] S. Perdikis, R. Leeb, J. Williamson, A. Ramsay, M. Tavella, L. Desideri, E.-J. Hoogerwerf, A. Al-Khodairy, R. Murray-Smith, and J. d. R. Millán, "Clinical evaluation of BrainTree, a motor imagery hybrid BCI speller," *J. Neural Eng.*, vol. 11, no. 3, p. 036003, 2014.

[2] S. Perdikis, R. Leeb, and J. d. R. Millán, "Context-aware adaptive spelling in motor imagery BCI," *J. Neural Eng.*, vol. 13, no. 3, p. 036018, 2016.

[3] S. Perdikis, L. Tonin, S. Saeedi, C. Schneider, and J. d. R. Millán, "The Cybathlon BCI race: Successful longitudinal mutual learning with two tetraplegic users," *PLoS Biol.*, vol. 16, no. 5, p. e2003787, 2018.

[4] I. Iturrate, R. Chavarriaga, L. Montesano, J. Minguez, and J. d. R. Millán, "Teaching brain-machine interfaces as an alternative paradigm to neuroprosthetics control," *Sci. Rep.*, vol. 5, p. 13893, 2015.

[5] P. W. Ferrez and J. d. R. Millán, "Simultaneous real-time detection of motor imagery and error-related potentials for improved BCI accuracy," in *Proc. 4th Int. Brain-Computer Interface Workshop and Training Course*, 2008, pp. 197–202.
